# Brain Processes Involved in Motor Planning Are a Dominant Factor for Inducing Event-Related Desynchronization

**DOI:** 10.3389/fnhum.2021.764281

**Published:** 2021-11-11

**Authors:** Kosei Nakayashiki, Hajime Tojiki, Yoshikatsu Hayashi, Shiro Yano, Toshiyuki Kondo

**Affiliations:** ^1^Department of Computer and Information Sciences, Graduate School of Engineering, Tokyo University of Agriculture and Technology, Tokyo, Japan; ^2^Biomedical Science and Biomedical Engineering, School of Biological Sciences, University of Reading, Whiteknights, Reading, United Kingdom

**Keywords:** brain-computer interface, EEG, grasping, isometric, event-related desynchronization

## Abstract

Event-related desynchronization (ERD) is a relative attenuation in the spectral power of an electroencephalogram (EEG) observed over the sensorimotor area during motor execution and motor imagery. It is a well-known EEG feature and is commonly employed in brain-computer interfaces. However, its underlying neural mechanisms are not fully understood, as ERD is a single variable correlated with external events involving numerous pathways, such as motor intention, planning, and execution. In this study, we aimed to identify a dominant factor for inducing ERD. Participants were instructed to grasp their right hand with three different (10, 25, or 40%MVF: maximum voluntary force) levels under two distinct experimental conditions: a closed-loop condition involving real-time visual force feedback (VF) or an open-loop condition in a feedforward (FF) manner. In each condition, participants were instructed to repeat the grasping task a certain number of times with a timeline of Rest (10.0 s), Preparation (1.0 s), and Motor Execution (4.0 s) periods, respectively. EEG signals were recorded simultaneously with the motor task to evaluate the time-course of the event-related spectrum perturbation for each condition and dissect the modulation of EEG power. We performed statistical analysis of mu and beta-ERD under the instructed grasping force levels and the feedback conditions. In the FF condition (i.e., no force feedback), mu and beta-ERD were significantly attenuated in the contralateral motor cortex during the middle of the motor execution period, while ERD in the VF condition was maintained even during keep grasping. Only mu-ERD at the somatosensory cortex tended to be slightly stronger in high load conditions. The results suggest that the extent of ERD reflects neural activity involved in the motor planning process for changing virtual equilibrium point rather than the motor control process for recruiting motor neurons to regulate grasping force.

## 1. Introduction

Brain-computer interface (BCI) technology has been extensively investigated over the last decade (Graimann et al., 2010; Do et al., 2011; Lebedev and Nicolelis, 2017). BCIs establish direct communication channels from the brain to control external devices. Especially, this technology is expected to facilitate neuro-rehabilitation for stroke patients, which is on the rise in an aging society. For example, BCI has been used to detect motor intention from brain activity and control medical/assistive devices such as exoskeleton or electrical stimulation to intentionally move paralyzed limbs (Tacchino et al., 2017; Bai et al., 2020). A widely used feature of brain signals to detect the motor intention is event-related desynchronization/synchronization (ERD/S) (Pfurtscheller and Lopes da Silva, 1999; Pfurtscheller, 2001), where ERD is a relative spectral power attenuation of electroencephalogram (EEG) during motor execution and motor imagery (Ron-Angevin et al., 2011; Formaggio et al., 2013), while ERS is a relative spectral power increase like a rebound mainly after the motor execution (Nakamura et al., 2021). Pioneering work by Takahashi et al. (2012) revealed that increasing the strength of functional electrical stimulation (FES) in response to the detection of ERD enabled the paralyzed ankle to bend spontaneously after the intervention, whereas the FES alone cannot induce such motor recovery of the stroke patients. This result indicates the critical function of closing the sensorimotor loop via the feedback loops between the motor intention and the somatosensory feedback. Thus, the successful BCI-based neuro-rehabilitation depends on accurate and reliable detection of motor intention, and closing of sensorimotor loops which can provide a feedback signal immediately after motor intention.

In order to detect motor intent from ERD, it is critical to elucidate the dominant neural processes underscoring motor execution reflected in ERD generation. Relevant candidates are “motor intention,” “motor planning,” and “motor command generation” (Kitazawa et al., 1997; Sober and Sabes, 2003, 2005; Desmurget and Sirigu, 2012). Motor execution can be performed via two distinct pathways. One pathway involves a feedforward-based control loop termed open-loop control. One such example is when intending to generate a given level of grasping force, motor plan is initially generated at upstream neural systems followed by a corresponding series of motor commands generated downstream in motor areas, thereby recruiting muscles in the motor units. In contrast, the other pathway involves a feedback-based control loop in which motor planning is adjusted to the real-time input from the limbs interacting with tools or the environment. The former control loop is unidirectional motor coordination after motor intention and planning are generated, which may be suitable for repetitive motion in a steady environment. Conversely, the latter requires the constant monitoring of the resultant components after motor execution and involves real-time regulation of motor planning.

Another point to note in the elucidation of ERD is that it occurs not only during exercise but also during motor imagery. Motor imagery has been widely used in the context of BCI training. Participants are typically instructed to imagine “execution of motion.” Previous studies have indicated that an increase in ERD strength is correlated with the success of imagining the kinetic aspects of motion such as strength of force and velocity of motion. Wang et al. recently reported that ERD induced by motor imagery may be modulated by the intensity of movement (Wang et al., 2017, 2019).

Most BCI studies have focused on training motor imagery in healthy participants. Nevertheless, this experimental protocol itself remains controversial, as participants must imagine moving their body parts yet remain motionless. The same issue holds for cases of paralyzed limbs; therefore, we hypothesized that motor imagery of healthy participants and motor attempts to move the paralyzed limbs are underpinned by different neurological pathways. In neuro-rehabilitation, stroke patients are engaged in motor attempts during therapeutic intervention. The neurological pathways involved should be similar to motor execution pathways in healthy participants. Therefore, we focused on training sessions of motor execution, rather than motor imagery in healthy participants.

In our previous study, we performed EEG experiments for studying the ERD during actual hand grasping with different motor loads (0, 2, 10, and 15 kgf) and grasping speeds (“Slow,” “Fast,” and “Hold” conditions in the paper) (Nakayashiki et al., 2014). We found that ERD was continuously generated during periodic execution (“Slow” and “Fast” conditions), while it tended to decrease when the participants keep their hand holding (“Hold” condition). Since ERD decreases even though it continues to exert exercise during maintenance, it is presumed that ERD does not directly reflect muscle strength exertion. On the other hand, Fry et al. (2016) reported ERD continues to occur in isometric movement. There are many differences in these experiments, but we focused the difference that our experiment was feedforward-controlled movement, while Fry's experiment was feedback-controlled movement. In the feedback control, the level of muscle strength exertion or hand position are displayed as visual feedback with respect to a target value (Kristeva et al., 2007); i.e., closed-loop visual motor control is performed in the brain, and errors from the target value are constantly fed back to the participants, and corrected. For the feedforward control, participants have to perform the task only by relying on tactile or somatosensory feedback (Gwin and Ferris, 2012). However, it is difficult for the participants to fine-tune the force output without visual feedback; thus, they adjusts the force based on the prior practice of the task. In this study, the ERD/S induced with or without real-time visual feedback of grasping force (i.e., feedback or feedforward control) is compared to investigate the effect of an error correction process in the brain on resultant ERD.

Another result of our previous studies suggested that grasping force and resultant ERD were not correlated. However, several ambiguities in the experimental procedure remained. For instance, since the strength of motor load was determined by choosing several hand-grippers, the grasping forces that the participants actually generated could not be confirmed. Therefore, in this experiment, an electronic grip dynamometer was used to measure the actual grasping force and to give the real-time feedback of grasping force level to the participants. In addition, the motor load was adjusted individually based on the maximum grip force of each participant.

In this study, we systematically investigate the effects of feedback and feedforward control on resulting mu and beta-ERD while participants are instructed to control grasping force. First, we investigate how the ERD can be generated when the closed-loop visuomotor control or feedforward control is imposed to participants. We hypothesize that the generation of mu-ERD reflects the motor planning rather than the motor intention, because it can be expected that the mu-ERD indicates the rebound during isometric contraction, same as “Hold” condition in our previous study (Nakayashiki et al., 2014). Second, we investigate the relationship between the grasping force level and resultant ERD amplitude. If the ERD is not related to the motor commands that would control the recruitment of muscle, it is expected that this relationship indicates no correlation.

## 2. Materials and Methods

### 2.1. Participants

Ten healthy male participants (aged 22–25 years, mean age: 23.1 years) took part in the experiment. All were right-handed, as assessed by the Edinburgh Handedness Inventory (Oldfield, 1971) and had no record of any neurological disorders. Participant recruitment and experimental procedures were approved by the ethics committee of the Tokyo University of Agriculture and Technology. Participants were informed of the aims and procedures of the experiment, and provided written informed consent prior to participation in the experiment.

### 2.2. Experimental Setup

**Figure 1:**
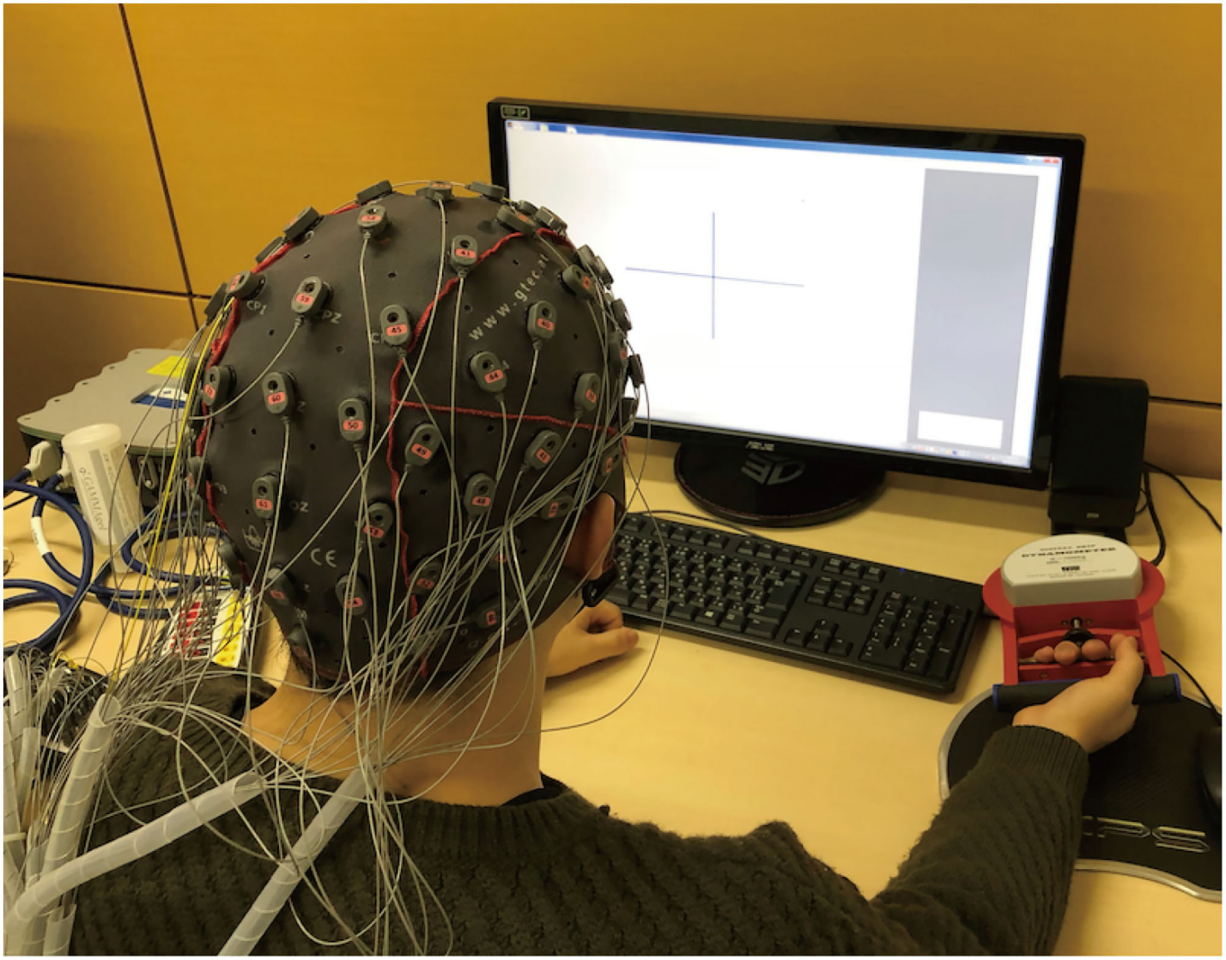
Experimental setup. Participants wore an EEG cap with 64-channel active electrodes (g.Tec Inc., Austria). Participants were seated in a comfortable chair and placed their right arm on a table such that a group of muscles of their upper limb were relaxed against gravity. An LCD monitor was located in front of them, enabling viewing of a visual cue on the display.

Participants were seated in a comfortable chair with their right arm placed on a table, such that their forearm muscles were relaxed against gravity (Figure 1). Participants were instructed to grasp a digital grip dynamometer (T.K.K.5710b, TAKEI Scientific Instruments Co., Ltd., Japan) with their right hand. Their grasping force level was measured using a strain amplifier (DMP-911B, Kyowa Electronic Instruments Co., Ltd., Japan) and stored in a personal computer. The grasping force data were analyzed online using original MATLAB and Simulink (MathWorks, MA) scripts to enable online visual force feedback. An LCD monitor was located in front of the participants to enable to present visual cues and grasping force level on the display according to the experimental condition.

During the experiment, participants wore an EEG cap with 64-channel active EEG electrodes (g.SCARABEO, g.tec, Vienna, Austria); the ground and reference electrodes were placed on the forehead (AFz) and left mastoids, respectively. The EEG signals were amplified using a digital bio-signal amplifier (g.HIamp, g.tec, Austria), and band-pass filtered between 0.1 and 100 Hz in the amplifier. Throughout the experiment, EEG activity was sampled at 512 Hz for the offline analysis.

### 2.3. Experimental Procedure

We designed an experimental paradigm to investigate how kinetic properties and sensory feedback conditions during grasping movement affected the resulting ERD. In the experiment, participants were instructed to grasp their right hand at one of three different force levels (10, 25, or 40%MVF: maximum voluntary force) under two visual feedback conditions: VF (with visual feedback of grasping force level displayed as the length of a red horizontal bar) or FF (without visual feedback, i.e., feedforward control). To avoid the effects of muscle fatigue, the following six experimental conditions were conducted in a fixed order for all participants, i.e., starting from the VF condition and 10%MVF, followed by the FF condition and 10%MVF, VF condition and 25%MVF, and so on. In each condition, participants were instructed to repeat the experimental tasks for 30 times. As participants were tasked to perform the VF condition first, this provided sufficient training opportunities to obtain self-regulatory ability of grasping force level in the subsequent FF conditions.

**Figure 2:**
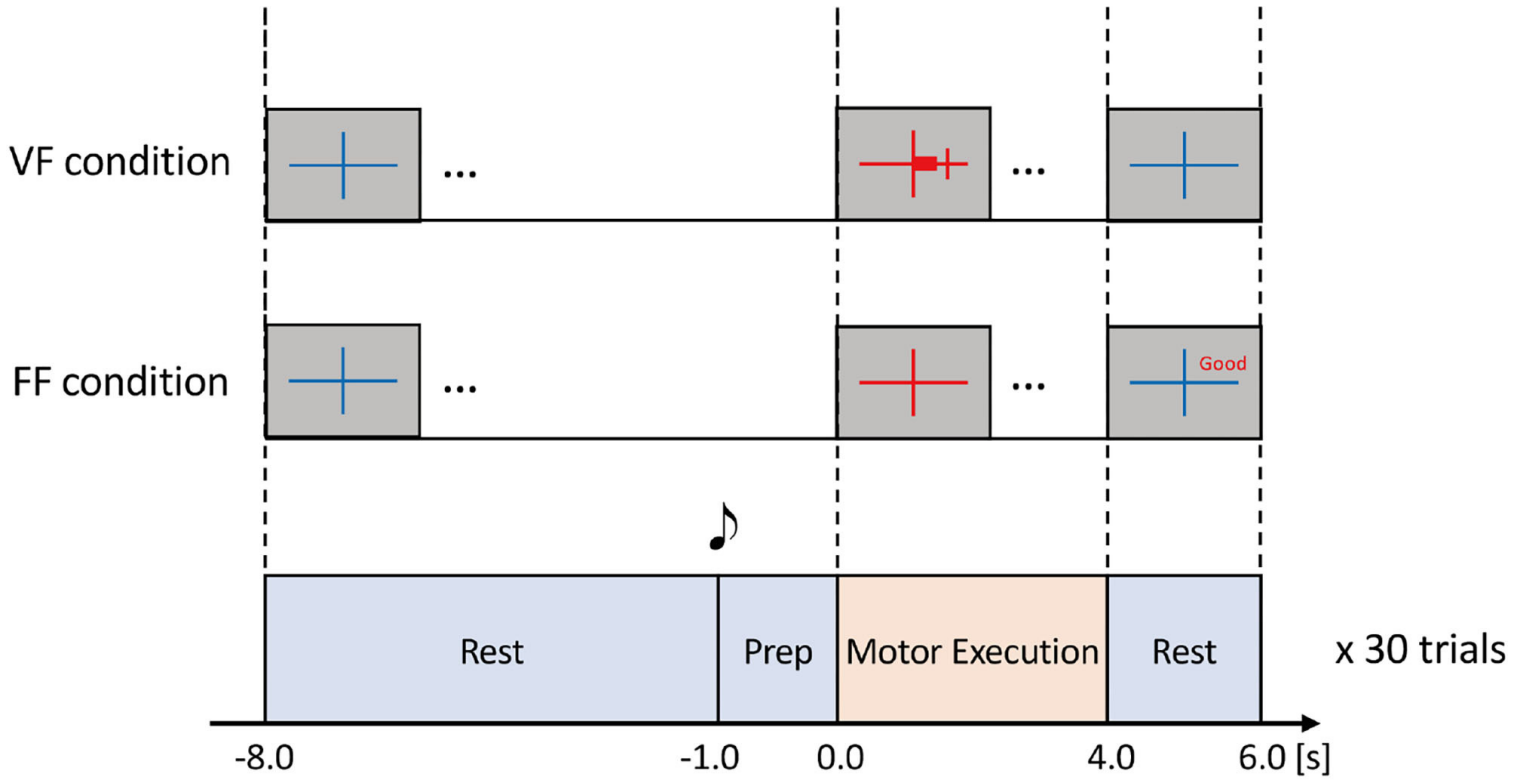
Experimental task. The task comprised Rest, Prep, and Motor Execution periods. During the Rest period, participants were instructed to relax. 1.0 s before the Motor Execution period, a beep sound was played for notification. During the Motor Execution period, the participants were instructed to grasp their right hand and maintain the grasping force within a specified range chosen from 10, 25, or 40%MVF with visual feedback (VF) or a feedforward (FF) condition. In the VF condition, a red horizontal bar indicated the extent of grasping force in real-time. In the FF condition, no online feedback was provided, but the precision of grasping force (i.e., the extent of error) was shown after the Motor Execution period. Thus, there were six experimental conditions according to the combination of %MVF × the way of feedback. In each condition, the experimental task was repeated 30 times to obtain sufficient EEG signals.

Each task consisted of Rest (10.0 s), Preparation (1.0 s), and Motor Execution (4.0 s) periods (Figure 2). During the Rest period, participants were instructed to relax while looking at a blue fixation cross on the screen. 1.0 s before the Motor Execution period, a beep sound was played to indicate the task initiation. During the Motor Execution period, the fixation cross turned red, and participants were instructed to grasp their right hand and maintain their grasping force level within a pre-specified range. Note that the participants had to keep clenching their hand for 4.0 s until the Motor Execution period was terminated (i.e., the fixation cross turned blue). In the FF condition; i.e., without the visual force feedback condition, verbal feedback was displayed on the screen for 2.0 s just after the Motor Execution period. For example, “Good” represented that the grasping force level was within the ± 10% range of the pre-specified target force level (see Figure 2). Other verbal expressions included “Too Strong” (20% or more), “Strong” (10% or more), “Too Weak” (−20% or less), and “Weak” (−10% or less).

### 2.4. EEG Signal Processing

We evaluated the time-course of event-related spectral perturbations to determine the modulation of ERD/S, according to each experimental condition.

The experimental trials in which the absolute EEG amplitude exhibited over 100 μ*V* were discarded from further analysis, as these signals were artifacts reflecting the eye-blink and/or body sway. The remaining EEG data were pre-processed using EEGLAB software (Delorme and Makeig, 2004). The acquired EEG data were subjected to 1 Hz high-pass filter, 40 Hz low-pass filter, and 50 Hz notch filter. EEG data were binned into 11.0 s epochs (−4.0 to 7.0 s) with respect to the onset of grasping force which aligned to 0.0 s. Epochs were visually checked and epochs that were estimated to have a lot of noise in them were removed. The Noise was confirmed in many epochs of the 40%MVF condition, and the majority of epochs were removed from some participants. Therefore, the 40%MVF condition data was considered inappropriate as EEG data and was excluded from the analysis.

We used Independent Component Analysis (ICA) as a method to remove artifacts in EEG, such as ocular electrograms, electromyograms, and body movements (Jung et al., 2000a). The ICA is a computational method for separating a multivariate signal into multiple independent components. By applying the ICA to the scalp electrode data, the activities at the signal sources can be estimated, and artifacts such as blinking can be separated from the estimated activity (independent components) at each signal source. In this study, denoising was adapted in the following manner. For each participant, EEG signals from four conditions were merged into a single data set, except for the 40%MVF condition. These data were adapted to *runica* function of EEGLAB using the infomax ICA algorithm (Bell and Sejnowski, 1995). If the topography, time course, or frequency spectrum of each independent component was inferred to reflect EMG, body motion, or eye movement artifacts, these component was excluded (Jung et al., 2000a,b; Allison et al., 2012), and the EEG signal for each channel was reconstructed with the remaining components.

Time-frequency maps were calculated using the wavelet transform. The ERD was calculated by normalization with respect to the time-averaged power during the most recent Rest period (i.e., −4.0 to −2.0 s).

### 2.5. Statistical Analysis

For the comparison of behavioral measures of grasping force across the three %MVF levels and two feedback conditions (VF and FF), a 3 × 2 two-way repeated measures ANOVA was conducted with the significance level sets at 0.01. As described in the EEG signal processing section, we excluded the artifacts from the recorded signals, because there was substantial amount of noise from the body motion in the EEG data under 40%MVF condition. However, we included the grasping force data under the 40%MVF condition into the statistical analysis to confirm the success or failure of the grasping force demonstration in the entire experiment.

Regarding the EEG data analysis, we chose a specific frequency band in which the strongest ERD was generated across each participant and condition, since the range of the frequency band for the ERD generation differs from participant to participant. After moving-averaging the intensity of the power spectra over the frequency at a width of 3 Hz, the frequency band with the strongest ERD at the beginning of the grasping (0.0–1.0 s) was calculated, and the ERD during the grasping (2.0–3.0 s) in that frequency band of interest was tested. Mu-ERD and beta-ERD were calculated for each frequency range of the mu band (8–13 Hz) and beta band (14–30 Hz). The calculated values of the ERD were summarized under the feedback and force level conditions, and statistically tested in the following manner; Shapiro-Wilk test was used to check whether the ERD of all participants (averaged by channel and condition) could be a normal distribution. If the results were rejected with the threshold of 0.01 in some cases, Wilcoxon signed-rank test was used for the statistical tests.

## 3. Results

### 3.1. Grasping Force

To clarify the relationship between the grasping force level and the EEG feature, we firstly analyzed the grasping forces that the participants actually exerted in the experiments.

**Figure 3:**
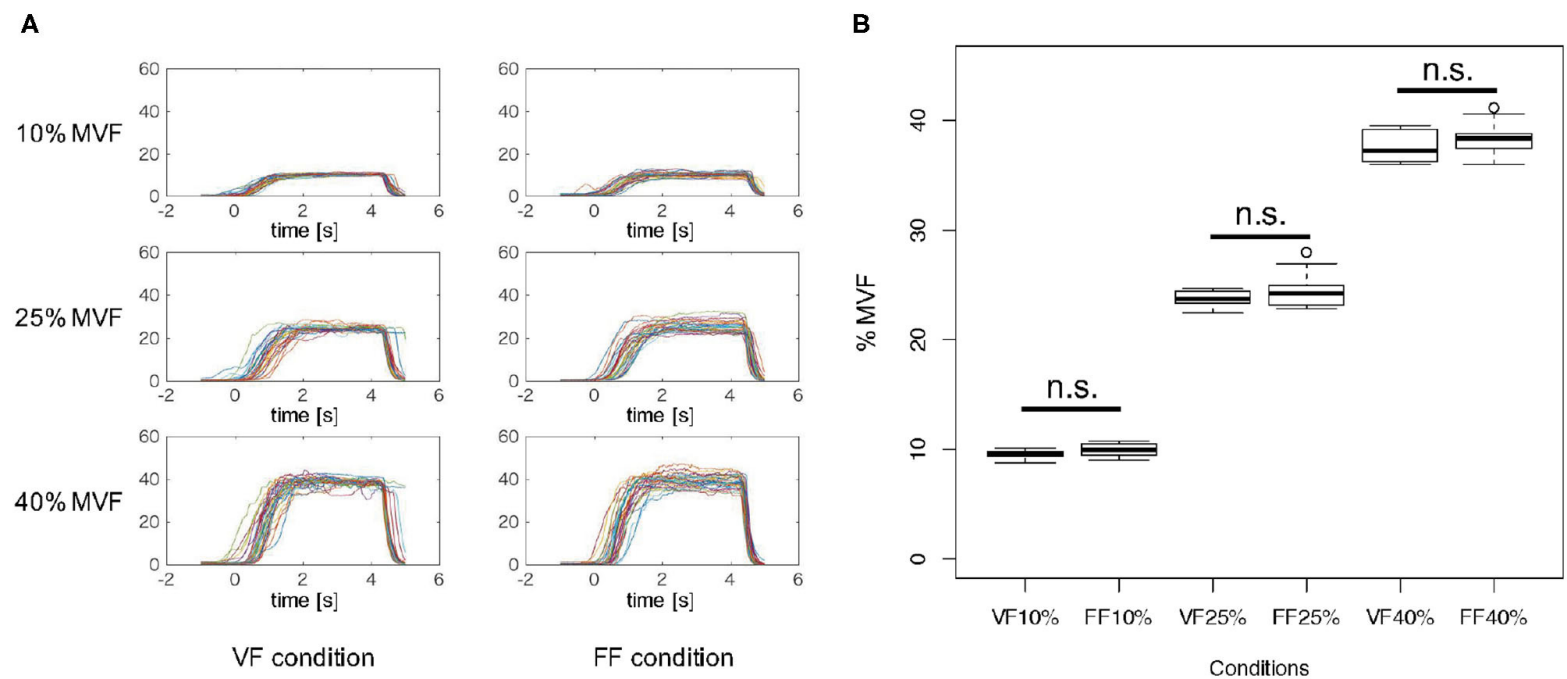
Grasping force in %MVF measure. **(A)** Grasping force time-series of a typical participant (Participant B) in %MVF measured under each experimental condition. Each graph includes 30 trials. As shown in these graphs, the grasping forces were maintained during 1.0–4.0 s, and a relatively large variability was observed in the FF condition. **(B)** Mean and standard error of grasping forces during 1.0–4.0 s across participants in each condition. Two-way ANOVA revealed a significant main effect of %MVF levels, but no significant difference was observed between feedback conditions. This implies that the participants exerted identical motor outputs in both VF and FF conditions.

Figure 3A indicates the time-series of grasping forces in %MVF measures of a typical participant (Participant B) under the FF and FB experimental condition. Each graph includes the trajectories of the resultant grasping force of 30 trials. As shown in Figure 3A, the grasping forces were maintained more or less precisely during the Motor Execution period, although relatively large variability was observed in the FF condition due to the lack of visual force feedback.

Figure 3B represents the mean and standard error of grasping forces during 1.0–4.0 s of the Motor Execution period across participants in each condition. To verify whether participants were able to produce the exact instructed level of force, we statistically analyzed the resultant grasping forces. A 3 × 2 two-way repeated measures ANOVA (*force level* × *feedback condition*) revealed a significant main effect on *force level* [*F*_(2, 18)_ = 8076.9, *p* < 0.01], but no interaction effect [*F*_(2, 18)_ = 0.4567, *p* = 0.54588] and significant main effect of *feedback condition* [*F*_(1, 18)_ = 2.3941, *p* = 0.1562] were noted. These results indicated that participants indeed exerted precise motor outputs according to the instructed force level in both FF and FB conditions.

Moreover, we investigated whether visual force feedback induced increase of frequency of grasping force adjustment. The %MVF between 2.0 and 4.0 s of the task period was extracted, and the number of zero-crossing with respect to the mean value was counted. A moving average of 16 points (about 0.03 s wide) was applied in order to eliminate fluctuations due to hand tremor and spinal reflex instead of control by visual and tactile force feedback. The median number of zero-crossing in all trials was calculated for each subject in each session, and Wilcoxon signed-rank test was performed under the VF and FF conditions. As a result, we found that the number of zero-crossing under the VF condition was significantly higher than that under the FF condition (*p* = 0.0312) (Supplementary Figure 1). These results suggested that participants performed a relatively large number of adjustments on their grasping force through the visual feedback. In addition, the comparison of the number of zero-crossing in the first half (15 trials) and the last half (15 trials) in the VF condition revealed there was no significant difference (*p* = 0.4161), leading to the conclusion that there is no learning effect in controlling the grasping force (Supplementary Figure 2).

### 3.2. Time-Frequency Map of the Event-Related Spectral Perturbation

**Figure 4:**
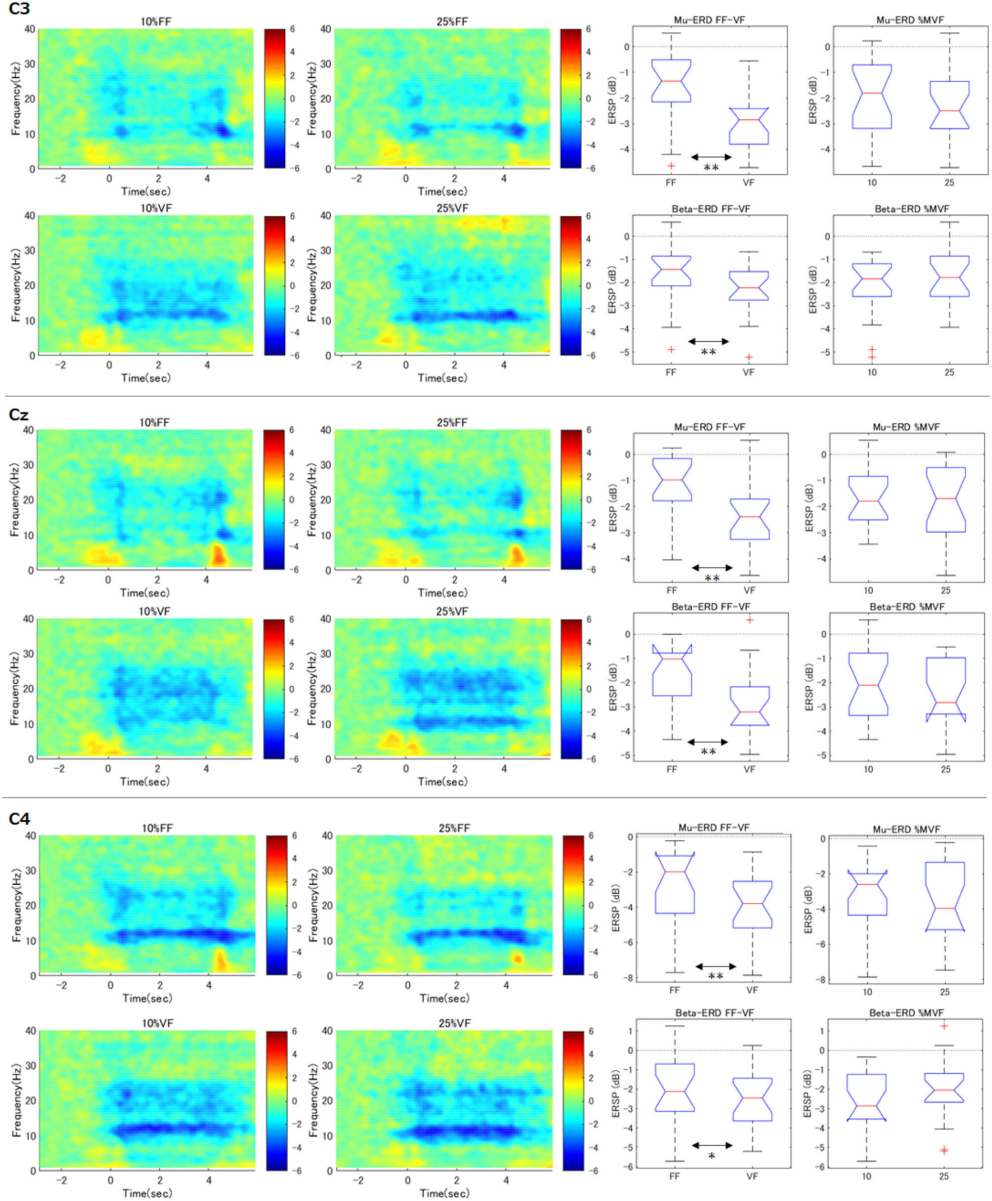
Time-frequency maps of ERD/S and comparison of ERD during maintenance of grasping (C3, Cz, C4). The graphs in the left two columns show the time course of relative power decreases (ERD) and increases (ERS) in the motor cortex under each condition. The horizontal axis is the time aligned with the beginning of the task period (0.0 s), and the vertical axis represents the frequency. The color bar indicates the relative power. In the FF condition, mu-ERD disappeared in the middle of the exercise execution period (1.0–3.0 s). The graphs in the right two columns demonstrate, for each participant and each condition, the ERD during the maintenance of grasping (2.0–3.0 s) was calculated and statistically processed for all participants (**p < 0.01). These results indicate that the mu-ERD in the VF condition was significantly stronger than that in the FF condition. On the other hand, there was no significant difference in mu-ERD between the force conditions. Beta-ERD showed the same trend as mu-ERD.

Time-courses of the relative power decrease (ERD) and increase (ERS) in primary motor cortex (C3, Cz, and C4) under each visual feedback and grasping force level condition were depicted in the left two column of Figure 4. The horizontal axis indicates the time aligned at the onset of the task period (0.0 s), and the vertical axis represents the frequency. The color bar indicates the relative power.

As can be seen in the results of contralateral hemisphere (C3) which would reflect right hand movements, apparent mu-rebound (i.e., disappearance of ERD in 8–13 Hz) was observed at the middle of the task period (1.0–3.0 s) in the FF condition (i.e., without visual force feedback), whereas mu-ERD was continuously confirmed in the VF condition.

### 3.3. Effects of Two Factors (Force and Feedback Conditions) at C3

To confirm the effect of the force levels and the feedback conditions on the resulting ERD at C3 channel, we statistically compared mu and beta-ERD in each condition (right two column in Figure 4). These notched box-plots represent the results of factor-by-factor testing of ERD during maintenance of grasping period (i.e., 2.0–3.0 s) calculated according to the process described in the statistical analysis section (**p < 0.01). For example, the graph of “Mu-ERD FF-VF” represents the comparison of mu-ERD in the FF and VF conditions without discriminating the grasping force levels (i.e., all the data of 10 and 25%MVF were used.). These results showed that mu-ERD was significantly stronger in the VF condition than that in the FF condition (*p* = 0.0003). On the other hand, there was no significant difference in mu-ERD between the “force condition” (*p* = 0.65). This trend was also true for beta-ERD, with a significant difference for “feedback condition” (*p* = 0.010) and no difference for “magnitude of force” (*p* = 0.062). In addition, no interaction between the two factors was confirmed (mu: *p* = 0.872, beta: *p* = 0.773). These results showed that ERD decreased during the exercise execution period only in the FF condition.

### 3.4. Effects of Two Factors in Surrounding Channels

**Figure 5:**
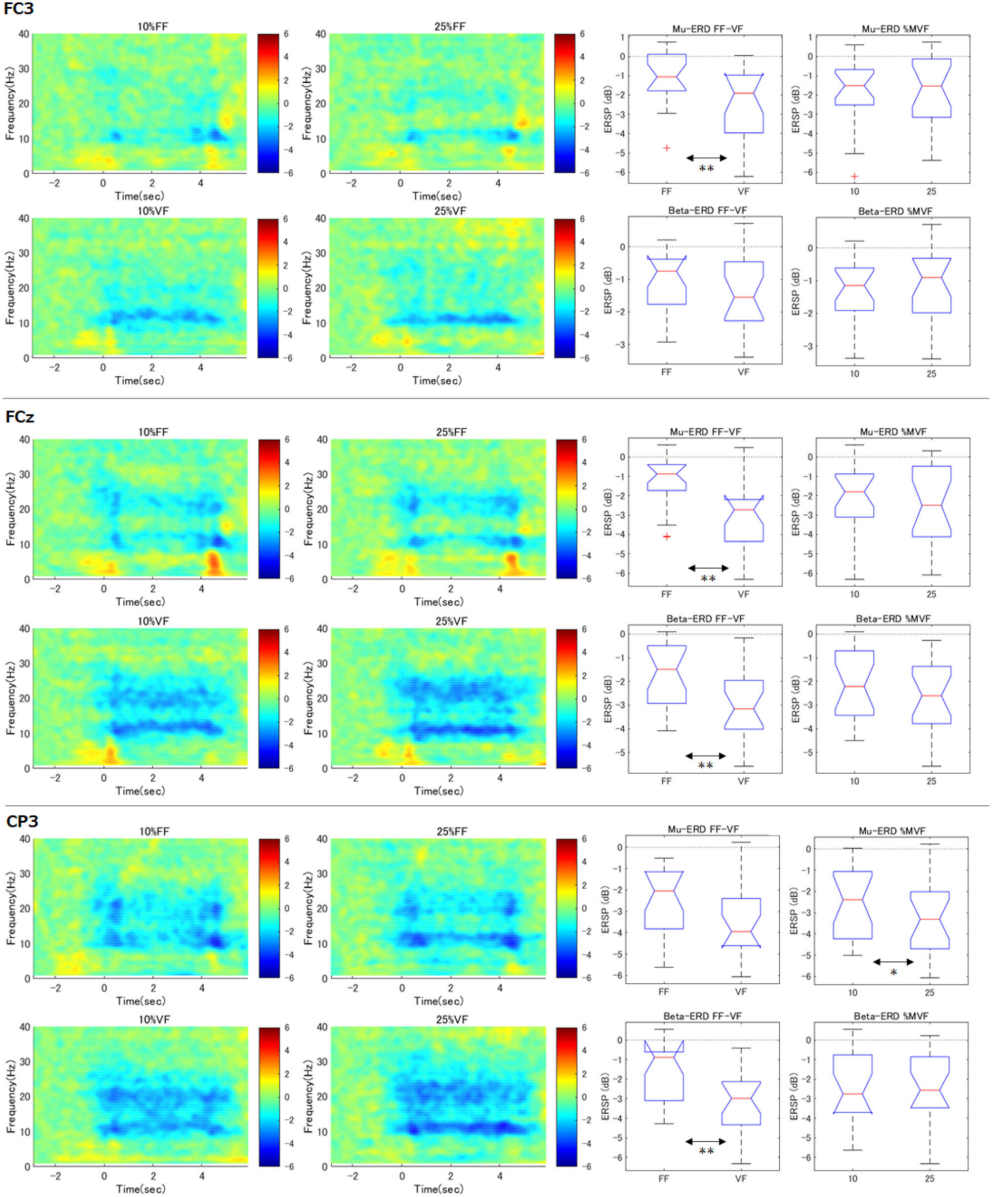
Time-frequency maps of ERD/S and comparison of ERD during maintenance of grasping (FC3, FCz, CP3). The same analysis as for C3 was performed for the surrounding channels. The trend of the tilt time-frequency map is similar to that of C3. Statistical tests on the resultant ERD during maintenance of grasping in FF condition showed that mu/beta-ERD for FCz, mu-ERD for FC3, and beta-ERD disappeared for CP3. only in CP3, mu-ERD occurred significantly more strongly when the load was strong (**p < 0.01, *p < 0.05).

Similar to right hand primary motor cortex (C3), we further analyzed the surrounding channels: Fz (supplementary motor cortex), FC3 (premotor cortex), C4 (left hand primary motor cortex), Cz (foot primary motor cortex), and CP3 (right hand somatosensory motor cortex) (see Figures 4, 5). As shown in the time-frequency map, in the FF condition, the mu-ERD of each channel tended to decrease in the middle of the task period (1.0–3.0 s). The statistical results were similar to those of C3 in FCz, Fz, C4, and Cz (both mu and beta-ERD were significantly stronger in the VF condition than in the FF condition). In FC3, mu-ERD tended to be significantly stronger in the VF condition as in C3, while beta-ERD showed a similar trend but no significant difference. In CP3, there was no significant difference in mu-ERD between the FF and VF conditions, while mu-ERD was significantly stronger in the 25%MVF condition than the 10%MVF condition. Beta-ERD tended to be significantly stronger in the VF condition as in C3. No interaction between the two factors (force and feedback) was observed in any of the channels.

## 4. Discussion

We investigated event-related spectral power decrease in the mu and beta frequency bands elicited by actual motion of the hand grasping under several force levels. In addition, by displaying the %MVF to the participants in real-time, we examined the effect of feedback or feedforward control on the ERD under maintaining grasping force. Experimental results showed that (1) grasping force level had no significant effect on the intensity of the mu and beta-ERD on motor cortex as a result of motor execution, and (2) online visual feedback of grasping force had a positive effect on inducing continuous mu-ERD.

Kilavik et al. (2013) reported that during stable object holding, beta oscillations display a relative increase in power and are phase-synchronized with the EMG of tonically contracting muscles. Stancák et al. (1997) used a finger lifting movement against several motor loads as the motor task, and reported that the duration of mu-ERD (not the time-averaged mu-ERD level) was significantly longer under the heaviest load condition and that post-movement beta-synchronization was also longer under the heaviest load compared to the no-load condition. The authors concluded that the ERD/S was influenced by the applied external load. Neurophysiological research by Tan et al. (2013) demonstrated that neural activity in sub-cortical areas was linked to motor effort. Furthermore, functional MRI studies indicated that cortical BOLD signal may correlate with grasping force levels (Cramer et al., 2002; Keisker et al., 2009). Pistohl et al. (2012) reported that the movement of a cup of two different weights performed by epileptic patients can be classified, albeit with low accuracy, by combining ECoG signals below 6 Hz and above 14 Hz. In their another study, they reported that ECoG of the human motor cortex could successfully distinguish two different grasping movements (precision vs. whole-hand grip) even if the weights of the manipulating objects were different (Pistohl et al., 2013). Wang et al. (2017) reported that motor imagery of different muscle strengths can be classified in real-time by beta-ERD but not mu-ERD. To summarize, in previous studies, there are some findings stating that fMRI and ECoG can distinguish the brain activities related to the weight of the load, but in the studies using EEG ERD, the tendency is different depending on the conditions. The different trends from our results where ERD in the motor cortex does not affect the level of muscle strength exertion are found in the results of Wang and Gwin. The major differences between those two experimental designs are motor imagery and foot movements. To our knowledge, the work that most closely resembles our results was reported by Chakarov et al. (2009) who indicated that EEG and EMG spectral power did not show any significant differences among the three force conditions, although the beta range EEG-EMG coherence increased as the load increased.

Although the above work appears controversial, the modulation of ERD may be dependent on several factors including (1) experimental paradigm, such as single execution or repetitive motion; (2) frequency range; (3) motor performance over task duration; and (4) brain region, including invasive or non-invasive approaches.

Human sensorimotor processing consists of sub-processes including motor intention, planning of motion trajectory, motor command generation, and receiving sensory feedback. Our findings demonstrated that ERD of the motor cortex may not reflect the strength of the motor load. We propose that the strength of mu and beta-ERD may reflect the motor planning process rather than motor command generation which recruits a group of motor neurons. On the other hand, our results also indicated that mu-ERD in the somatosensory cortex may reflect the intensity of the motor load; Mu-ERD in the somatosensory area may reflect the strength of the skin sensation that changes depending on the strength of the load. However, our result is different in terms of the frequency band and location of the channels found from the results of Wang, Gwin, and Pistohl, who found differences in brain activity with motor load introduced in the previous section (Gwin and Ferris, 2012; Pistohl et al., 2012; Wang et al., 2017). This may indicate that the processing of the load may differ, depending on the type of exercise. The difference may stem from the fact that motor imagery was used in Wang's experiment while motor execution was used in our experiment, thus the brain processes for the both might be different with each other. In addition, it is still a question of how to prove if subjects were able to imagine different grasping forces by motor imagery. Gwin also reported that the extent of ERD correlates with the force level, but they only compared the ERD immediately after the muscle exertion in both isometric and isotonic conditions. Therefore, the brain processes may differ between instantaneous and continuous exertion of force. Furthermore, Pistohl used ECoG for motion classification experiment, and the channel range they analyzed included not only the motor cortex but also the somatosensory area. If the classification result comes from the information in the area, their finding is consistent with our result that the strength of the load is related to the mu-ERD of the somatosensory area rather than the motor area. However, signals of 5 Hz or less and 54 Hz or more mainly contribute to classification accuracy, these are considered to be a neural process different from mu and beta-ERD.

Our experimental results demonstrated that online visual force feedback during grasping movement has positive effects on inducing the mu and beta-ERD. Similar results have been reported in previous studies. There was no visual feedback that allowed the foot position to be fine-tuned. Their experimental conditions and results are similar to the FF condition in our study. In this experiment, isometric wrist flexion movement was performed, and the amount exerted was visually feedback in real-time. As a result, it has been reported that beta-ERD continued to occur during movement. Their experimental conditions and results are similar to the VF condition in our study. They reported that premotor cortex and parietal lobe BOLD are activated by visual feedback compared to without visual feedback during maintenance of grasping. Although not exactly in line with the brain regions in our study, EEG has lower spatial resolution than fMRI Considering this point, Mayhew's physiological finding seems to be similar to our result. From these results, in our study, in the case of no visual feedback while controlling the grasping force, mu-ERD was probably produced in correlation with motor intention or a few times motor planning for kinematic control of virtual equilibrium points. In the case of real-time visual force feedback, participants can monitor the difference between the current grasping force and target value of the force, and they continue to adjust the grasping force, thereby controlling their virtual equilibrium points. It is plausible that the continuous planning of virtual equilibrium points resulted in continuous mu-ERD generation. As a concern, in this study, the order of experimental conditions was fixed in order to avoid fatigue. In particular, the VF condition is a practice for the FF condition, and it cannot be denied that learning of tactile force control may affect the ERD. However, since there was no significant difference in the number of zero crosses in the first and the last half of VF trials, it is presumed that visual force feedback is still dominant in the last half and the influence of the learning effect is low.

Now, let us discuss the possibility of applying BCI-based neuro-rehabilitation. As discussed in the Introduction, the neurological pathways of motor execution in healthy participants are presumed to be similar to the motor training of patients, as patients with hemiparetic conditions actually attempt to move their body parts, thereby, generating the motor intention and the motor planning. Thus, our study with the focus on motor execution may contribute to the development of the BCI applications for the training of patients. Monitoring the mu-ERD during the motor training and the continuous ERD generation indicated that the real-time control of the target posture involved the re-planning of body posture/kinematics. Future studies should investigate how the re-planning of body posture monitored by BCIs can benefit recovery of motor coordination. Since our experimental paradigm was conducted only on healthy young participants, in the future study, it is necessary to confirm if the same principal of closing the sensorimotor loops applies to paraplegics and the elderly.

## Data Availability Statement

The raw data supporting the conclusions of this article will be made available by the authors, without undue reservation.

## Ethics Statement

The studies involving human participants were reviewed and approved by the Ethics Committee of the Tokyo University of Agriculture and Technology. The patients/participants provided their written informed consent to participate in this study. Written informed consent was obtained from the individual in Figure 1 for the publication.

## Author Contributions

KN supervised the study and designed the experiment. KN and HT developed the experimental system, performed the experiments, and statistical analysis. KN and TK contributed to discussion of the obtained results. KN, TK, and YH wrote the manuscript. SY provided fruitful comments for the experimental procedure and analysis. All authors have read and approved the final manuscript.

## Funding

This research was supported by JSPS KAKENHI (Grant Numbers: JP17KK0064, JP18K19732, JP19H05727, JP20H02111, and JP20J10024) and a research grant from the Institute of Global Innovation Research at TUAT.

## Conflict of Interest

The authors declare that the research was conducted in the absence of any commercial or financial relationships that could be construed as a potential conflict of interest.

## Publisher's Note

All claims expressed in this article are solely those of the authors and do not necessarily represent those of their affiliated organizations, or those of the publisher, the editors and the reviewers. Any product that may be evaluated in this article, or claim that may be made by its manufacturer, is not guaranteed or endorsed by the publisher.
