# Supplementary figures and images for: Brain Processes Involved in Motor Planning Are a Dominant Factor for Inducing Event-Related Desynchronization
Source: Front Hum Neurosci. 2021 Nov 11;15:764281. doi: 10.3389/fnhum.2021.764281 (PMC8631820; doi:10.3389/fnhum.2021.764281)

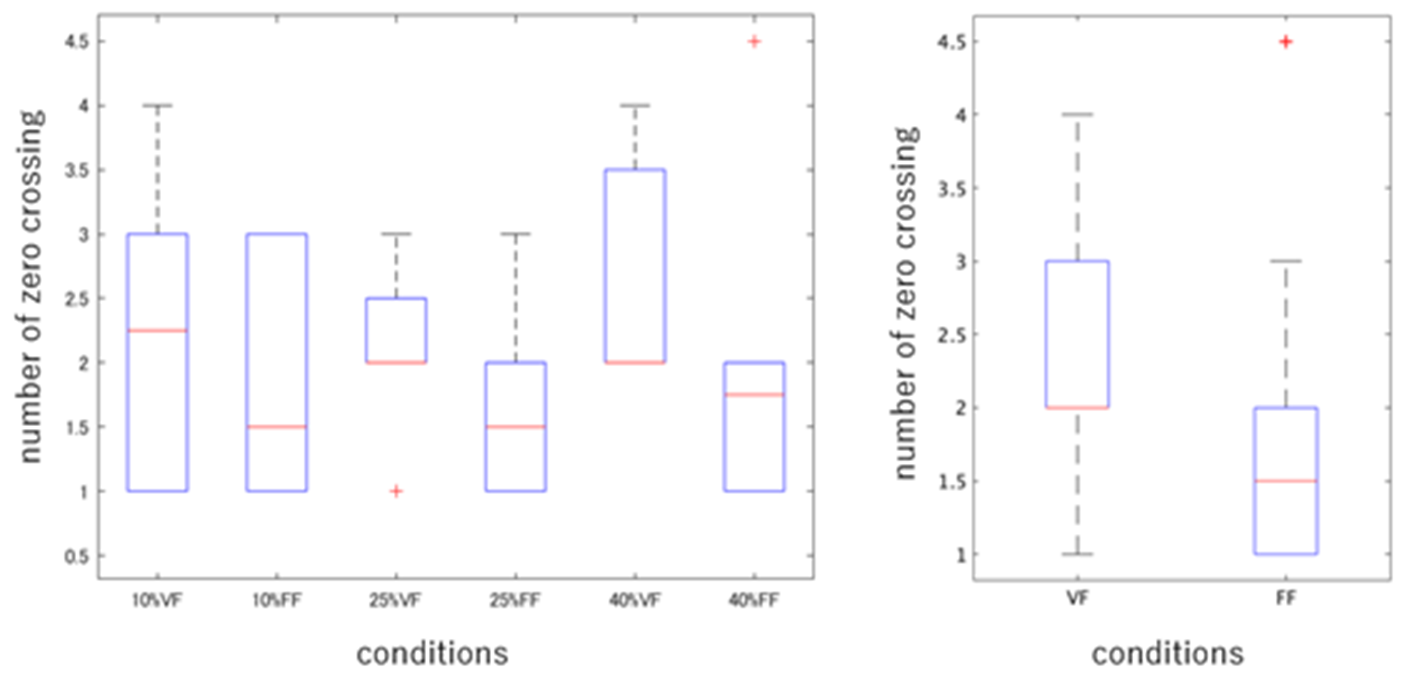

Supplement: Supplementary file 1 [file Image_1.TIF]

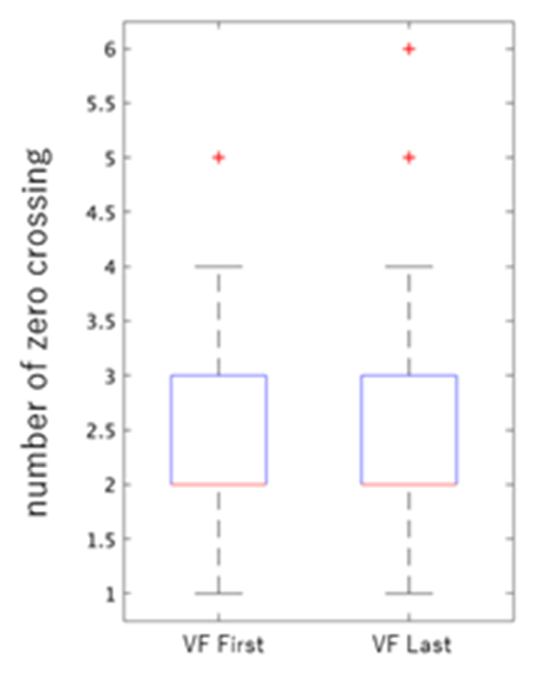

Supplement: Supplementary file 2 [file Image_2.TIF]
